# Effect of a multimodal continuing professional development initiative on HIV testing in primary care in British Columbia, Canada

**DOI:** 10.1186/s12981-024-00688-3

**Published:** 2024-12-19

**Authors:** Brenna M. Lynn, Jennifer A. Barrows, Vivian W.Y. Lam, Vernon R. Curran

**Affiliations:** 1 Faculty of Medicine, Division of Continuing Professional Development (UBC CPD), University of British Columbia, Vancouver, Canada; 2 Faculty of Medicine, Division of Population Health and Applied Health Sciences, Faculty of Medicine, Division of Continuing Professional Development, Memorial University of Newfoundland, University of British Columbia, Vancouver, Canada

**Keywords:** HIV, Continuing medical education, Physicians, Primary care, Program evaluation

## Abstract

**Introduction:**

Many persons are unaware of HIV infection until they present in an advanced stage of the disease. Diagnosing HIV infection in its earliest stages reduces morbidity and mortality and contributes to improved public health. Increased testing for HIV is critical for prevention, and primary care providers play an essential role in early HIV screening. However, lack of knowledge and confidence are barriers to the adoption of screening practices. Continuing professional development (CPD) may enhance greater HIV testing uptake amongst primary care providers by improving awareness and comfort. This paper aims to report on the impact of a multimodal CPD initiative to increase HIV screening and test ordering across primary care settings in British Columbia, Canada.

**Methods:**

The ‘HIV Testing Initiative in Family Practice’ was designed as a multimodal education strategy to encourage family physicians to adopt routine HIV testing in their practices. The initiative encompassed a variety of core and supplementary educational activities, including interactive in-person CPD workshops, practice resources, and patient education materials. An interrupted time series study was undertaken to evaluate the effect on HIV test ordering before and after workshop participation. Participants also completed post-workshop surveys to assess satisfaction.

**Results:**

In total, 316 individuals participated in the core educational activities of the initiative. The number of HIV tests ordered increased significantly amongst participants following workshop participation (*p* < .001). HIV test ordering increased for both rural and urban providers with no significant difference (*p* = .075) on the number of tests ordered between these groups. Participants were very satisfied with workshop participation, with an overall mean satisfaction rating of 4.78 (out of 5) and reported high satisfaction with the format (4.64 out of 5) and interactivity (4.76 out of 5) features across the workshops.

**Discussion:**

The findings suggest that the introduction of a multimodal CPD intervention may have effected change in family physicians’ HIV testing practices. The HIV testing rates of physicians who had participated in CPD workshops increased significantly, and participant evaluation data indicated a greater understanding of the rationale for routine HIV testing, as well as an increase in comfort with recommending HIV tests to patients.

## Introduction

Advances in human immunodeficiency virus (HIV) treatment have changed our understanding of HIV infection as a chronic, manageable condition, with many people able to live healthy, long, and active lives [1, 2]. Unfortunately, many persons who are unaware of their infection remain undiagnosed until they present in an advanced stage of HIV or with an AIDS-related condition [3]. Undetected HIV and late diagnosis are associated with ill health, increased risk of death and onward viral transmission [4]. Diagnosing HIV infection in its earliest stages reduces morbidity and mortality and contributes to improved public health by reducing the period an individual might unknowingly spread the virus [5, 6]. It has been estimated that over one-third of new transmissions may be traced to individuals unaware of their HIV infection [7].

Regrettably, many individuals diagnosed with late HIV infection are often not tested for HIV despite having had multiple contacts with their healthcare system [8, 9]. Screening for HIV is recommended across many national guidelines in the United States, Canada, and the United Kingdom [6]. Since 2006, the US Center for Disease Control and Prevention (CDC) has recommended that providers across various healthcare settings perform routine HIV screening for most individuals aged 13 to 64 [7, 10]. Primary care providers are essential in screening for HIV infection [9, 11, 12]. In Canada, Health Canada’s HIV Screening and Testing Guide gives family physicians an essential role in assessing patients at risk of HIV infection, detecting those who are infected, and recommending treatment options [5]. Increasing the offer of HIV testing is believed to be a pivotal way to facilitate early diagnosis and treatment, reduce the burden of illness, and delay the progression of the disease.

According to Chin et al. [2], more systematic approaches to HIV screening in primary care settings offer greater potential to improve overall diagnosis. General practice-based HIV screening can lead to increased and probably earlier diagnosis of HIV, which enhances the length and quality of life of people who are HIV positive and might reduce onward transmission [6]. There is increasing evidence that HIV testing is acceptable to patients in a primary care environment, operationally feasible and may reduce social stigma associated with the disease [9]. However, the rates of HIV testing have been suboptimal, particularly in primary care settings, with many providers frequently missing opportunities to test for HIV, not offering testing at all, or not making the diagnosis when patients present to them with suggestive symptoms [3, 7, 9, 13].

Numerous studies have reviewed barriers to physician HIV testing [10, 11, 13–17]. Physicians have reported multiple factors interfering with routine HIV screening and testing, including underestimating patients’ risks, lack of understanding about guidelines and the benefits of early HIV diagnosis, competing clinical priorities, and uneasiness in having sexual history discussions with patients. A lack of experience and inadequate HIV education and training have been reported as critical concerns among primary care providers who have not previously tested patients for HIV [9, 16].

Growing evidence suggests that promoting awareness of HIV testing and educating primary care providers about the benefits of HIV testing is essential for better uptake of HIV testing [18, 19]. Continuing professional development (CPD) involves supporting healthcare workers to keep up to date with best practices and address deficits in their knowledge and practice [20]. CPD programs have been shown to be effective in increasing physicians’ uptake and adherence to clinical guidelines for chronic diseases [21]. Research has also demonstrated an association between knowledge and barriers to HIV testing, including HIV testing rates, as well as the positive effect of CPD on improving HIV screening outcomes [8].

Several systematic reviews have reported that CPD which is more interactive, incorporates multiple educational techniques, and involves repeated exposures leads to more positive learning outcomes, including knowledge and skill gain [22–24]. Mansouri and Lockyer [25] found that CPD using multiple methods resulted in larger effect sizes on physician performance and patient health outcomes, while Davis and Galbraith [24] found that using multiple techniques of instruction and multiple-exposures had a more significant overall positive effect than single technique or single-exposure CPD. Studies reporting more interactive methods such as case discussion, role-play, or hands-on practice sessions are also generally more effective and lead to more significant improvement in physician performance and patient health [22–24].

This paper reports on the impact of a multimodal CPD initiative in British Columbia, Canada, that aimed to increase HIV screening and test ordering practices across primary care settings.

## Methods

### HIV testing initiative in family practice

The HIV Testing Initiative in Family Practice was designed as a multimodal education strategy to encourage family physicians in British Columbia (BC) to adopt routine HIV testing in accordance with best practice recommendations. The goals included supporting family physicians in learning why, how, and when to test for HIV, facilitating the integration of routine HIV testing into clinical practice, and linking physicians to resources for connecting patients who test positive to quality care. The initiative encompassed a variety of core and supplementary educational activities and resources offered across rural and urban communities in British Columbia, Canada, between April 2013 and September 2016. Core educational activities included highly interactive in-person accredited CPD workshops, educational lunch sessions, and sessions for postgraduate medical residents. Supplementary educational activities encompassed resources physicians could access at their convenience, such as e-articles, a website hub, and an e-learning module (Table 1). The central core educational activity was interactive small-group workshops primarily targeted at family physicians. The workshop content was tailored for each community, and workshops were facilitated by local family physician champions with practices focused on HIV care and management. HIV testing resources in multiple languages were also made available to workshop participants, as well as other resources to support practice change and manage positive test results.

**Table 1:** Summary of multimodal HIV testing initiative in family practice

Core Educational Activities	
*Workshops*	Interactive small group workshops primarily targeted to family physicians.
*Lunch Sessions*	One-hour accredited educational sessions offered in family practice/clinic locations covering the rationale and evidence for routine HIV testing, resources for implementing routine testing and an overview of initial patient management after a positive test result.
*Resident Session*	One-hour session offered to postgraduate medical residents with resource packages on HIV testing.
**Supplementary Educational Activities/Resources**	
*E-learning Module*	An e-learning module titled ‘HIV Testing in Primary Care’ was developed covering: •New HIV Testing Guidelines •Special Considerations for Testing and Plan for Testing •Result Guidelines •Initial Management of an HIV Diagnosis
*HIV Testing Reports*	Physicians had the option of signing up to receive personalized ‘HIV testing report cards’ on a quarterly basis via e-mail containing aggregated data on number of HIV tests ordered.
*This Changed My Practice e-article*	Online resource offering brief e-articles on pearls or practice tips. An e-article titled ‘ HIV in Family Practice: testing & diagnosis’ was published to raise awareness of the new recommendations for HIV testing.
*Website Hub*	The ‘hiv.ubccpd.ca’ was a central resource for family physicians on HIV testing, providing access to current information and recommendations.

### Evaluation study

An interrupted time series study was undertaken to evaluate the effect of the multimodal educational initiative on family physicians ordering of HIV tests. Data on HIV test ordering was obtained from the British Columbia Centre for Disease Control (BCCDC) and Provincial Health Services Authority (PHSA) laboratories. The British Columbia HIV testing guidelines recommend that healthcare providers know the HIV status of all patients under their care [26]. For the purpose of this study, HIV test ordering is defined as the number of HIV tests ordered by the family physician and processed by community laboratories. An increase in HIV test orders indicates that the patient went to a laboratory following an appointment with their family physician and an HIV test was carried out. HIV test ordering data was analyzed for consenting study participants at six months pre- and six months post-workshop participation. Participants were divided into two groups to create an experimental and control group. The experimental group included participants who attended the workshop early in the study period. In contrast, the control group comprised participants who participated in the workshop later (similar to a waitlist control group design). HIV testing data for the control group came from a time period (7 months to 1 year) before this group of participants attended the workshop. HIV testing data for the control group was compared with data for the experimental group for 12 months (e.g., six months pre- and six months post-workshop) using two-way repeated measures ANOVA.

Workshop participants also completed post-workshop surveys to evaluate satisfaction with participating in the workshops and learning benefits. The survey questionnaire included several items assessing participants’ satisfaction with format (presentation, discussions, audience size, etc.), job relevance (relevance to my practice/patients), compatibility (compatibility with my expectations), interactivity (adequate opportunities for interaction), and overall rating of the workshop. Items were rated using a scale of “1 = Unsatisfactory to 5 = Exceptional”. Ethical approval was received from the Behavioural Research Ethics Board, University of British Columbia.

## Results

In total, 316 individuals participated in the initiative’s core educational activities, with 25% of these participants representing rural primary care providers. Table 2 summarizes the average monthly number of HIV tests ordered (i.e. HIV tests processed by the laboratory) for the experimental and control study groups from 6 months before to 6 months after workshop participation.

**Table 2:** Mean monthly HIV test ordering pre and post-workshop participation – experimental and control study groups

		Experiment (*N* = 79)		Control (*N* = 79)	
		Mean	Std. Deviation	Mean	Std. Deviation
**Pre-workshop**	T-6	2.38	3.52	2.16	2.58
	T-5	3.00	4.73	1.78	2.68
	T-4	3.03	3.59	2.43	3.32
	T-3	2.90	4.44	1.92	3.48
	T-2	3.86	5.76	2.12	2.61
	T-1	4.84	9.17	1.99	2.61
**Post-workshop**	T + 1	6.90	9.71	1.99	3.47
	T + 2	7.71	12.72	2.06	2.92
	T + 3	7.32	13.96	1.99	2.72
	T + 4	6.59	10.27	2.19	3.57
	T + 5	6.66	9.66	2.18	3.28
	T + 6	6.38	8.25	2.29	2.82

*Time T + 1 represents month in which participants attended workshop

**Fig. 1:**
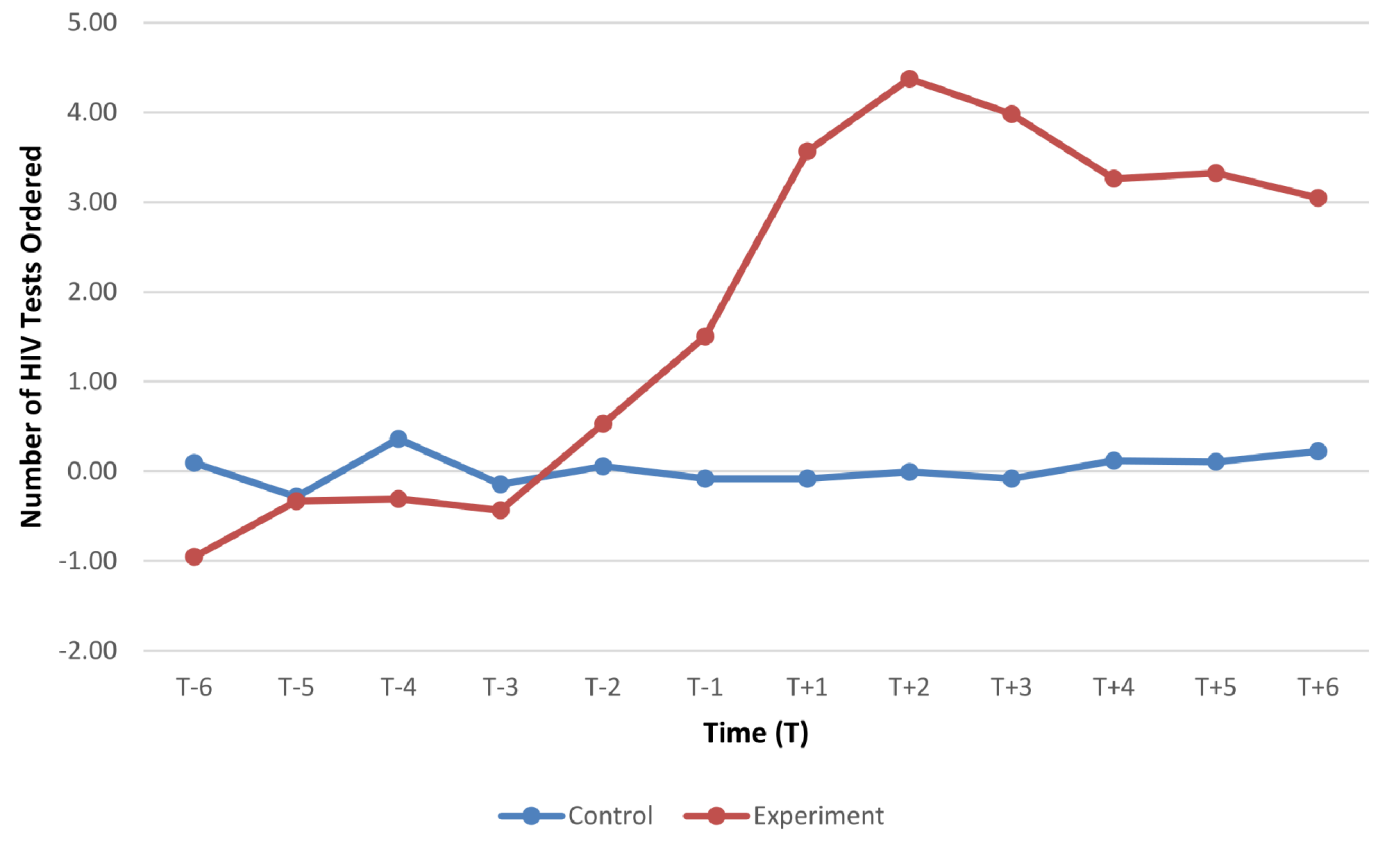
Line chart summary of mean monthly HIV test ordering pre and post-workshop participation – experimental and control study groups *Time T+1 represents month in which participants attended workshop

Repeated measures ANOVA of pre- and post-workshop HIV test ordering for the experimental and control groups showed a statistically significant group effect, F(1, 156) = 13.748, *p* < .001. The main effect of time was also statistically significant, F(2.578, 402.206) = 9.325, *p* < .001, and there was a significant group time interaction, F(2.578, 402.206) = 9.209, *p* < .001, indicating that the number of HIV tests ordered changed over time between the experimental and control groups. The number of HIV tests ordered after the workshop increased over time amongst experimental study group participants compared with the control group, as shown in Fig. 1 below.

**Fig. 2:**
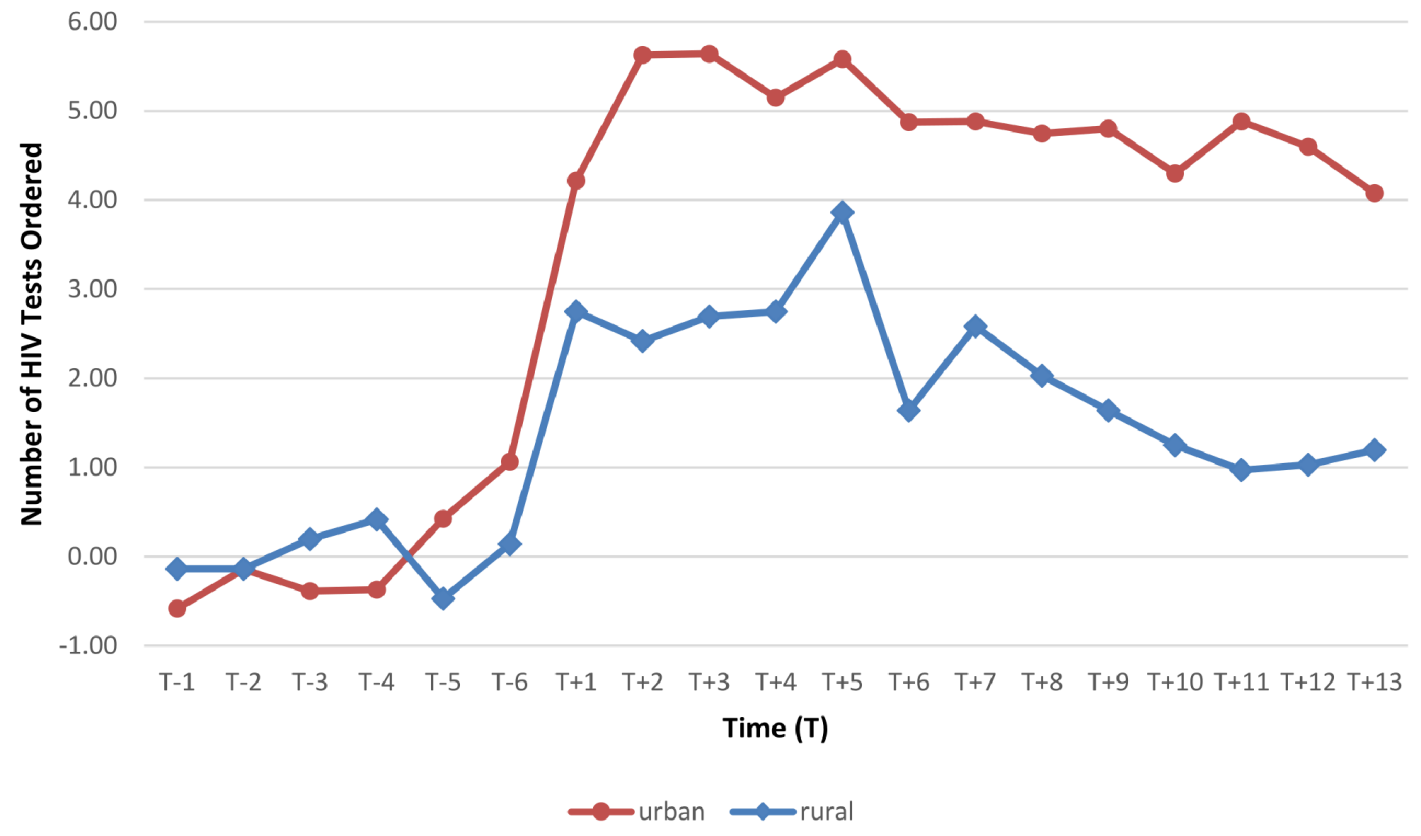
Line chart summary of mean monthly HIV test ordering pre and post-workshop participation – urban vs. rural study participants *Time T+1 represents month in which participants attended workshop

Figure 2 shows the number of HIV tests ordered over 19 months for rural and urban family physicians participating in the CPD workshops. The line chart indicates increased test ordering following participation for both rural and urban providers.

Table 3 also summarizes the mean monthly test ordering for study participants who attended workshops in rural versus urban locations for six months before and after workshop participation.

**Table 3:** Mean monthly HIV test ordering pre and post-workshop participation – urban vs. rural

		Urban (*N* = 142)		Rural (*N* = 18)	
		Mean	Std. Deviation	Mean	Std. Deviation
Pre- workshop	T-6	4.14	7.31	1.06	1.55
	T-5	3.48	4.86	0.61	1.54
	T-4	2.75	3.85	0.94	1.59
	T-3	2.67	3.18	1.28	1.45
	T-2	2.92	4.11	0.89	1.28
	T-1	2.49	3.34	0.78	0.94
Post-workshop	T + 1	7.26	9.56	3.94	6.36
	T + 2	8.69	13.86	3.44	6.34
	T + 3	8.76	16.82	3.28	7.71
	T + 4	8.20	15.68	3.83	7.21
	T + 5	8.63	14.43	4.94	9.77
	T + 6	7.87	13.75	3.17	6.56

*Time T + 1 represents month in which participants attended workshop

A two-way repeated measures ANOVA was used to compare changes in HIV testing between the rural and urban physician participants for a period of 12 months (i.e., six months pre-workshop and six months post-workshop). The results indicate a statistically significant effect of time, F(1.749, 276.421) = 5.460, *p* < .05. The effect of group approached significance but was, however, not statistically significant, F(1,158) = 3.217, *p* = .075, indicating the number of HIV tests ordered by participants did not differ between the rural and urban participants.

Participant evaluation surveys were also administered for workshops (*n* = 17) conducted between January 2013 and December 2016. In total, 244 survey responses were received from workshop participants, and respondents’ self-reported gender were 44% male (*n* = 82) and 56% female (*n* = 105), and the majority of respondents included family physicians (*n* = 143) and specialist physicians (*n* = 10). Other survey respondents included nurse practitioners/NPs (*n* = 6), registered nurses/RNs (*n* = 3), and public health nurses/PHNs (*n* = 4). Most respondents also practiced in mainly urban locations of Metro Vancouver (*n* = 88) and the Lower Mainland (*n* = 42). Table 4 provides a summary of satisfaction ratings. Overall mean satisfaction with the format and interactivity features across the workshops were 4.64 and 4.76 respectively, while satisfaction with the overall relevance of the learning was 4.58.

**Table 4:** Participants’ satisfaction with workshops

Workshop	*N*	Q1. Format Mean (*n*)	Q2. Job relevance Mean (*n*)	Q3. Compatibility Mean (*n*)	Q4. Interactivity Mean (*n*)	Q5. Overall rating Mean (*n*)
Workshops A (A1, A2)	25	4.58 (24)	4.50 (24)	4.50 (24)	4.71 (24)	4.70 (23)
Workshops B (B1, B2)	16	4.63 (15)	4.69 (16)	4.63 (16)	4.69 (16)	4.69 (16)
Division Workshops (D1–D6)	134	4.55 (110)	4.58 (109)	4.65 (109)	4.45 (110)	4.69 (108)
Workshop W1	18	4.41 (17)	4.35 (17)	4.65 (17)	4.65 (17)	4.71 (17)
Workshop W2	14	4.58 (12)	4.67 (12)	4.75 (12)	4.83 (12)	4.83 (12)
Workshop W5	8	4.63 (8)	4.13 (8)	4.63 (8)	5.00 (8)	5.00 (8)
Workshop W6	10	4.60 (10)	4.50 (10)	4.60 (10)	4.70 (10)	4.60 (10)
Workshop W7	15	4.90 (15)	4.90 (15)	4.87 (15)	4.90 (15)	4.90 (15)
Workshop W8	4	4.90 (4)	4.90 (4)	4.87 (4)	4.90 (4)	4.90 (4)
**All Workshops**	**244**	**4.64**	**4.58**	**4.68**	**4.76**	**4.78**

## Discussion

To increase HIV screening and testing in primary care, a number of authors support the idea that clinicians should be trained to be more proactive and confident in addressing HIV testing [2, 3, 7]. According to Martínez Sanz et al. [8], training primary care providers in HIV prevention and screening is essential and can change routine HIV screening practices. Education about HIV topics may reduce healthcare provider concerns and discomfort about having sensitive conversations with their patients by improving their knowledge and skills. The goals of such training may include increasing awareness of guidelines, dispelling misperceptions of patient resistance to HIV testing, and increasing efficacy for conversations related to sexual histories [7].

The ‘HIV Testing Initiative in Family Practice’ was designed as a multimodal education strategy to foster increased adoption of routine HIV testing. The initiative encompassed a variety of core and supplementary educational offerings. Core educational offerings included interactive, in-person accredited CPD workshops. Supplementary educational offerings encompassed resources accessible at the participants’ convenience, such as e-articles, a website hub, and an e-learning module. HIV testing materials in multiple languages were also made available to workshop participants and other enabling resources to support practice change. The findings from the study suggest that participation in HIV testing workshops and the possible exposure to other aspects of the initiative and resources may have affected change in physicians’ HIV testing practices. The results show an effect of time in that more HIV tests were ordered and family physicians sustained this practice over time with current and new patients. HIV testing rates by physicians who had participated in the workshops demonstrated substantial and sustained increases, and participant evaluation data indicated a greater understanding of the rationale for routine HIV testing, as well as an increase in comfort with recommending HIV tests to patients. Although the number of HIV tests ordered was higher for urban participants than in rural regions, the increase in testing post-workshop between the two groups approached but was not significant. This suggests that workshop participation may have effectively changed physicians’ HIV testing practice and indicates a possible difference due to location. A potential factor influencing the difference between the urban and rural groups could be related to the effect of stigma. People living in rural communities often report and experience more HIV stigma than urban communities, while Canadians living in rural communities are less likely than their urban counterparts to be knowledgeable about HIV/AIDS or to talk about it [27]. During the time this multimodal education strategy was being implemented, urban-based hospitals in British Columbia had active campaigns to promote HIV testing through media and poster strategies which may have normalized the idea of routine HIV testing for patients living in urban-based settings. Additionally, it is well documented that people living in rural communities report greater barriers to accessing care which reduces the rates of health service utilization [28] and this factor may have also negatively impacted patients’ ability to access HIV testing through a laboratory. Another possible factor contributing to lower HIV testing in rural settings is family physicians tend to have more diverse practice models in rural medicine [29]. Rural physicians are considered generalists who may work in acute and long-term care settings in addition to their family practice. Therefore, given the diverse nature and scope of practice, the opportunity to order more HIV tests in family practice as a rural physician may be lower compared to urban-based family physicians who tend to have a more homogenous family practice taking place predominately in primary care clinics.

Interestingly, a review of new HIV diagnoses in British Columbia between 2008 and 2017 published by the British Columbia Centre for Disease Control does indicate that the rate of new HIV diagnoses in the year 2017 (3.8 per 100,000 population) decreased to its lowest point since 2003, when HIV became reportable [30]. HIV testing yield reflects the percent positivity rate which is the proportion who test positive for HIV out of the total number of people tested. Unfortunately, the HIV testing yield data from family practice settings was not available for the study. As a surrogate marker, HIV testing yield was measured in acute care British Columbia hospital settings during the time this study took place and HIV testing yield was reported to be 6/1000 in that 30 positive HIV cases were detected when 5000 people were tested for HIV [31]. Family physicians participating in the workshops reported that by increasing routine HIV testing in their family practicet hey had diagnosed a number of HIV positive cases that otherwise may have not been discovered.

The evidence around CPD suggests that providers who participate in formal CPD activities are more likely to provide better care than their peers who do not participate [32]. CPD, which is designed to be interactive, practice-based, and longitudinal, is also believed to yield better outcomes [33]. Multicomponent interrupted CPD interventions appear to be more effective than single interventions and show more significant evidence of increased effect [24]. For example, workshops or learning activities held at different points may allow learners time and opportunity to process and apply new knowledge in the practice setting before reinforcement through subsequent learning experiences. Davis and Davis [33] also suggest that there is good evidence that interactive CPD experiences foster more positive learning outcomes by encouraging greater interplay between participants and participants with instructors. Practice enablers, such as patient education materials, also support translating knowledge into practice. The ‘HIV Testing Initiative in Family Practice’ was developed and implemented around these principles of multimodal and interactive CPD design. The study findings suggest such design elements may have been a contributing factor to program success in enhancing and sustaining HIV test ordering among primary care providers.

A primary limitation of the study was the inability to pinpoint which specific core and supplementary learning methods, resources, or tools may have been most impactful on physicians’ learning and actual changes in practice. It is also unclear how other activities, resources, or initiatives beyond participation in the CPD programming may have influenced knowledge of HIV testing and practice change. Nonetheless, the findings support other studies involving multimodal CPD approaches. Kang et al. [19] found that an intensive multimodal training program for family physicians using group learning and interactions with expert HIV clinicians appeared to increase the number of HIV-positive patients seen and the number of combination antiretroviral therapies prescribed. Leber et al. [4, 34] randomized controlled study of an HIV educational outreach program promoting screening in general practice found increased and earlier HIV diagnosis and an improved testing uptake of 45%. Martínez et al. [8] also found a higher HIV testing rate among primary care providers who had participated in HIV training and higher HIV diagnosis rates amongst those reporting improved knowledge. Future research might explore the aspects of multimodal CPD that have the greatest effect on HIV screening and testing practices, as well as the influence of in-person versus online components and resources on knowledge, confidence and skill gains.

## Data Availability

No datasets were generated or analysed during the current study.
